# Otosurgery with the High-Definition Three-Dimensional (3D) Exoscope: Advantages and Disadvantages

**DOI:** 10.3390/jcm10040777

**Published:** 2021-02-16

**Authors:** Małgorzata Wierzbicka, Witold Szyfter, Grażyna Greczka, Wojciech Gawęcki

**Affiliations:** Department of Otolaryngology and Laryngological Oncology, Poznań University of Medical Sciences, 60-355 Poznań, Poland; otosk2@gmail.com (M.W.); otosk2@ump.edu.pl (W.S.); grazynagr@interia.pl (G.G.)

**Keywords:** stapedotomy, tympanoplasty, cholesteatoma, chronic otitis media, surgery, exoscope, 3D imaging

## Abstract

Background: The aim of the study was to describe our initial experience with the high-definition three-dimensional (3D) exoscope for middle ear surgery versus the operating microscope. Methods: The study included 60 randomly chosen patients diagnosed with otosclerosis (*n* = 30) or chronic otitis media (*n* = 30) with a clinical indication for surgery. The primary measurement was the subjective estimation of quality of the visibility of the operating field provided by the 3D exoscope—VITOM-3D (Karl Storz, Tuttlingen, Germany) in comparison to the operating microscope. Results: All procedures, except for two (3.3%) converted to the microscope, were successfully completed using a 3D exoscope. In both stapedotomy and tympanoplasty, the exoscope was superior to the microscope during more superficial portions of the procedures. By contrast, in deeper areas of the middle ear, the exoscope provided significantly worse visibility, but usually not suboptimal. Both intraoperative bleeding and the narrow surgical field substantially reduced the visibility with the 3D exoscope in comparison to the microscope. Conclusions: Overall, our study shows that the 3D exoscope offers excellent, highly magnified, and well-illuminated high-definition images of the surgical field. However, our experience revealed several important limitations of this system, including decreased depth perception in deep areas of the tympanic cavity and reduced visibility in a difficult surgical field, with subsequent need to switch to an operating microscope in select cases.

## 1. Introduction

Advances in intraoperative optics and new surgical visualization systems such as the three-dimensional (3D) exoscope system have recently been reported as a viable alternative to surgical microscopes and endoscopes [1]. The 3D exoscope is used in a similar manner to a standard operating microscope without a wide field of view, with dual image sensors for 3D visualization. Although operating microscopes provide excellent illumination and magnification, their use is limited to two people: a surgeon and an assistant. Consequently, other members of a surgical team (and residents in training) are limited to viewing the images on monitors that present only a limited view of the depth of the surgical field and anatomical details. By contrast, the 3D exoscope can project stereoscopic 3D images to an external monitor, which allows for all observers in the operating theatre (wearing 3D glasses) to see the same high-quality images visualized by the operating surgeon [2,3,4]. It is especially valuable in university settings to improve the learning experience of surgical residents [5]. The exoscope is an established tool for neurosurgical visualization, providing images on a display monitor similar to an endoscope but positioned externally to the operating field like a microscope [2,3,4,5,6,7,8]. To date, only a limited number of published reports have described the use and feasibility of the 3D exoscope in head and neck procedures, mainly for postauricular transcortical mastoidectomy [9,10] and lateral skull base surgery [4,6] but also in cochlear implants surgery [10].

In this context, we carried a cross-sectional analysis to assess the feasibility of using the 3D exoscope to perform stapedotomy in otosclerosis and tympanoplasty in chronic suppurative otitis media. In this observational study, we evaluated the quality of subjective visibility with the 3D exoscope perceived by an operating surgeon and compared it to the operating microscope at all the stages of both surgical procedures.

## 2. Experimental Section

### 2.1. Study Design

This was a prospective cross-sectional analysis conducted in an academic, tertiary referral center. The regional ethics committee deemed this study to be exempt. The study included 60 randomly chosen patients diagnosed with otosclerosis (*n* = 30) or chronic suppurative otitis media with cholesteatoma or granulation (*n* = 30) with a clinical indication for surgery. All the patients underwent video-assisted surgery with the 3D exoscope, which was performed between January 2018 and December 2018. All stapedotomy procedures were performed by a single surgeon (WS, experienced with over 3000 stapedotomies with a microscope). The tympanoplasty procedures were performed by two surgeons (WS and MW, both with over 2000 procedures with a microscope). Both surgeons are right-handed, and they have more than 20 years of experience in otosurgery. An endaural approach (with an incision of skin between tragus and helix and with use of retractors) was used in all the patients undergoing stapedotomy. In all the tympanoplasty cases, an incision was made behind the auricle. All stapedotomies were performed under local anesthesia and neuroleptoanalgesia. All tympanoplasties were performed under general anesthesia.

### 2.2. Inclusion and Exclusion Criteria

There were no separate inclusion and exclusion criteria for the patients analyzed in this study. Every month, a few of the adult patients admitted to the Department and qualified for the stapedotomy or tympanoplasty were randomly chosen and operated on with the exoscope. Thus, the research group consisted of patients with varying degrees of technical difficulties.

### 2.3. Groups’ Characteristics

The otosclerosis group was comprised of 23 women (76.7%) and 7 men (23.3%), with a mean age of 37.3 years (range, 24 to 54). Of these, 22 patients (73.3%) underwent an initial stapedotomy while the remaining eight underwent stapedotomy in a second ear (26.7%). The tympanoplasty group included 18 women (60.0%) and 12 men (40.0%), with a mean age of 42.8 years (range from 23 to 59). In 24 patients, tympanoplasty was performed for the first time (80.0%), and in 6 (20.0%) it was a second-look procedure. Among the 30 patients with chronic suppurative otitis media, 26 were with cholesteatoma and 4 with granulation tissue. According to the European Academy of Otology and Neurotology (EAONO)/Japanese Otological Society (JOS) Joint Consensus Statements on the Definitions, Classification, and Staging of Middle Ear Cholesteatoma [11], we had 3 patients in stage 1, 22 in stage 2, and 1 in stage 3.

### 2.4. Equipment Description

**Figure 1:**
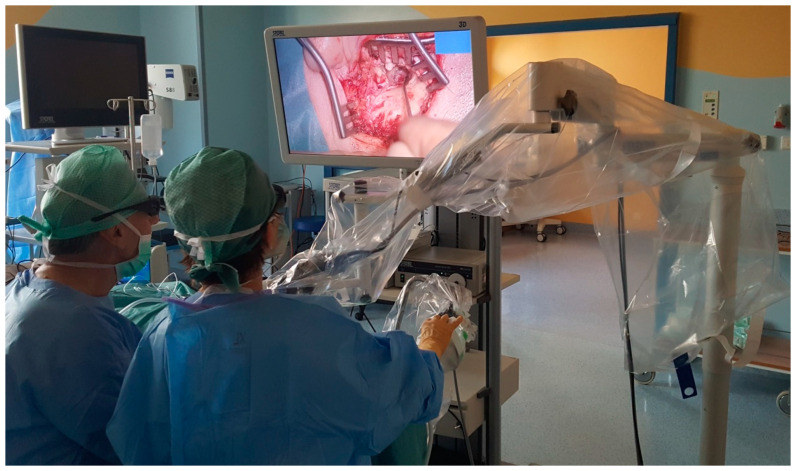
Middle ear surgery with the 3D exoscope—left ear, retroauricular incision, tympanomeatal flap preparation.

All the analyzed patients underwent video-assisted surgery with the VITOM 3D, a high-definition exoscope (Karl Storz, Tuttlingen, Germany) (Figure 1). The images from a standard operating microscope (VARIO MPS 88, Karl Zeiss, Jena, Germany) were used as reference points to assess visibility with the 3D exoscope.

### 2.5. Assessment Tools

The operating surgeons compared the quality of visibility with the exoscope to their prior recollection of visibility through the microscope from thousands of other middle ear surgeries. In order to make their subjective assessment, the surgeons filled in a form at each stage (stages 1–10) intraoperatively, and the subjective comparison was based on the following scale: (0) both instruments are considered comparable; (−1), the microscope is considered superior, and (+1) the exoscope is considered superior. During all the surgeries, the microscope was also prepared to be used at any single moment in case a surgeon needed to confirm his/her subjective assessment or to convert from the 3D exoscope to the operating microscope.

In stapedotomy, the various surgical stages are relatively consistent across most cases. For the purposes of this study, we defined and assessed the following stages: the initial image of the operating field (stage S1); ease of tympanomeatal flap formation (S2); ease of opening the tympanic cavity (S3); ease of widening the external auditory canal (S4); visualization of the incudostapedial joint (S5); assessment of the oval window area (S6); ease of removal of the stapes superstructure (S7); facial nerve assessment (S8); footplate assessment and perforation (S9); and prosthesis placement maneuver (S10).

The surgical stages for tympanoplasty, by contrast, can vary from patient to patient. Therefore, we assessed only the main stages of tympanoplasty, which had to be assessable for all operations. All cases included cholesteatoma or granulation tissue removal. The following stages were assessed: initial view of the operating field—planum mastoideum (stage T1); posterior meatal wall skin incision + tympanic membrane assessment (T2); antromastoidectomy (T3); cholesteatoma or granulation removal form mastoid process (T4); lateral semicircular canal visualization (T5); cholesteatoma or granulation removal form tympanic cavity (T6); cholesteatoma or granulation removal form hidden areas (protympanum and/or sinus tympani) (T7); temporal fascia harvesting (T8); ossiculoplasty (T9); and myringoplasty (T10).

### 2.6. Outcome Measures

The primary measurement was the comparison of the surgeons’ subjective assessments of the quality of the visibility of the operating field provided by the 3D exoscope and the operating microscope at each stage and for the whole procedure in both types of surgery. Furthermore, we evaluated the impact of intraoperative conditions on the visibility with the exoscope. What is more, we analyzed the necessity for the conversion from the 3D exoscope to the microscope. The conversion was defined as the moment when the visibility with the exoscope was suboptimal due to bleeding, difficult anatomy, or a narrow operating field, and an experienced surgeon did not have an operational sense of surgical confidence and decided to switch to the microscope, which he/she had known for decades. Additionally, we assessed the duration of the procedure (minutes).

### 2.7. Statistical Analysis

Statistica software (v.13, TIBCO Software, Palo Alto, CA, United States) was used to perform the statistical analysis. The test of proportion was used to compare the visibility provided by the exoscope with the one provided by the microscope throughout the procedure and in the particular steps of the surgery. Due to the lack of a normal distribution for the total visibility variable, the Mann–Whitney test was used for comparison of normal and difficult operating conditions (intraoperative bleeding and narrow operating field). The level of statistical significance was set at *p* < 0.05.

## 3. Results

### 3.1. Stapedotomy

The mean duration of the procedure was 41.3 min (range, 20 to 70), and it did not differ significantly from the average time it took for this procedure to be performed with the microscope in our department by the same surgeon (40 min). In all the cases, surgery and healing were uneventful. All 30 procedures were completed with the 3D exoscope without requiring a switch to the operating microscope. As shown in Table 1, the 3D exoscope provided better visibility of the surgical field in the first six stages (a positive sum of points). In stages S1–S4, the differences were statistically significant. By contrast, in stages S7–S10, the microscope provided statistically significantly better visibility. Although the visibility with the exoscope was rated worse in S7–S10, it was not suboptimal. Thus, the conversion to the microscope was not required and surgery was completed with the exoscope in all the cases.

**Figure 2:**
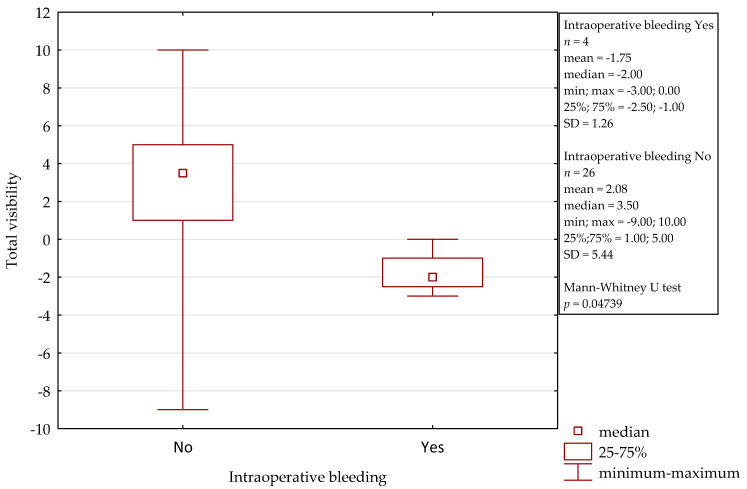
Influence of intraoperative bleeding on the visibility with the 3D exoscope during stapedotomy.

**Figure 3:**
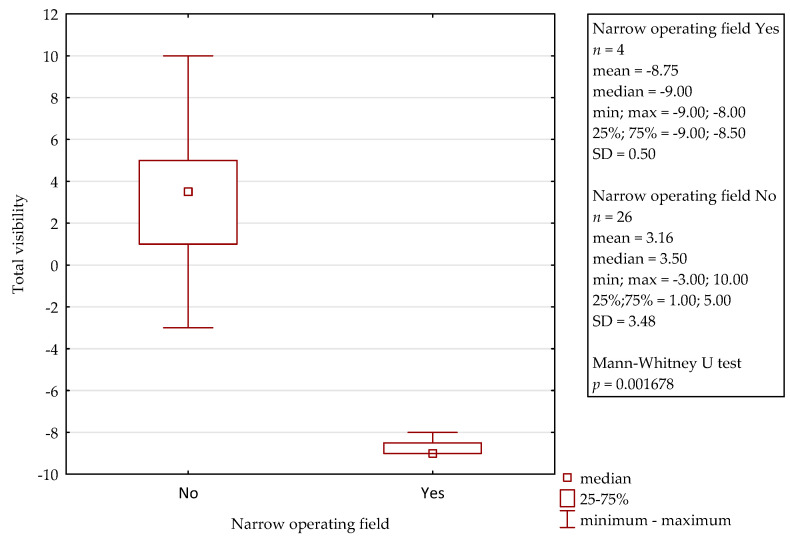
Influence of the narrow operative field on the visibility with the 3D exoscope during stapedotomy.

**Figure 4:**
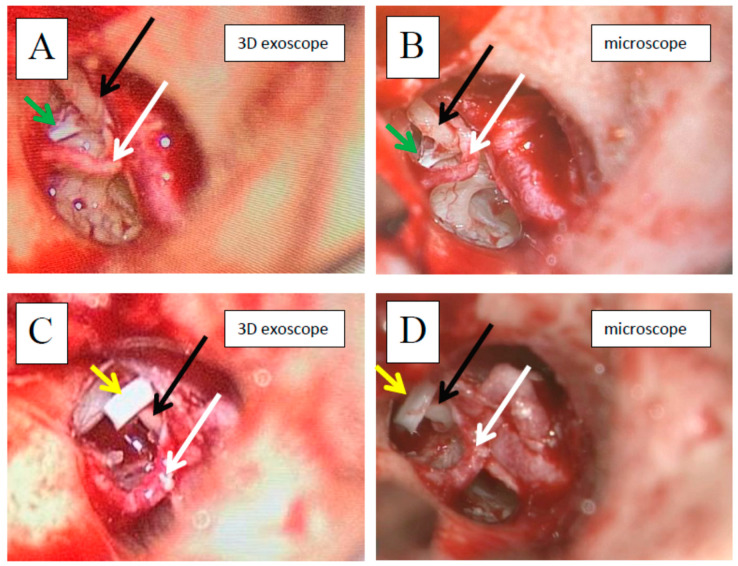
Intraoperative view during stapedotomy (right ear, endaural approach); **A** and **B**—tympanic cavity after tympanomeatal flap elevation, **C** and **D**—tympanic cavity after suprastructure of stapes removal and stapes prosthesis insertion; **A** and **C**—with the exoscope, **B** and **D**—with the microscope; long process of incus (black long arrow), chorda tympani (white long arrow), stapedial muscle tendon (green short arrow), stapes prosthesis (yellow short arrow). Images shown are 2D representations.

In comparison to the microscope, the 3D exoscope was evaluated as better in 48%, as comparable in 19.7%, and as worse in 32.3% for the whole procedure (after the summation of the results from all the stages). The differences were not statistically significant (*p* = 0.4884). The sum of the obtained points for all the stages of all the surgeries was 47, with a mean of 1.57 and a median 3.00 for one complete procedure. Furthermore, there were some evident differences concerning the influence of intraoperative conditions. Both intraoperative bleeding (observed in four cases) and the narrow surgical field (found in another four cases) significantly reduced the visibility with the 3D exoscope in comparison to the microscope. The details are presented in Figure 2 and Figure 3. An intraoperative view during stapedotomy, both with the exoscope and the microscope, is presented in Figure 4.

### 3.2. Tympanoplasty

The mean duration of the procedure was 78 min (range, 45 to 130) and it did not differ significantly from the average time it took for this procedure to be performed with the microscope in our department by the same surgeons (76 min for cholesteatoma stage 2). In all the cases, surgery and healing were uneventful. Most (28/30; 93.3%) of the tympanoplasty procedures were completed with the 3D exoscope. However, the conversion to the operating microscope was necessary in two cases, both due to bleeding during cholesteatoma and granulation removal from the tympanic cavity and hidden areas of the middle ear (protympanum and/or sinus tympani) (stages T6/T7). As shown in Table 2, the 3D exoscope provided better visibility of the surgical field in the first five stages (T1–T5) and in reconstructive stages at the end of the surgery (T8–T10). In stages T1–T3 and T8, differences were statistically significant. By contrast, in stages T6 and T7, the microscope provided statistically significantly better visibility. The visibility with the exoscope was rated worse in T6–T7, but except for two cases, it was not suboptimal. Thus, the exoscope was exchanged for the microscope only in these two cases, and in the remaining patients, the surgery was completed with the exoscope.

**Figure 5:**
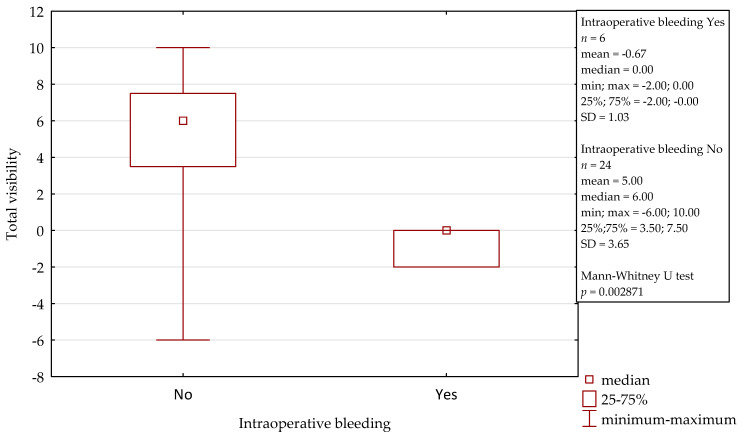
Influence of intraoperative bleeding on the visibility with the 3D exoscope during tympanoplasty.

**Figure 6:**
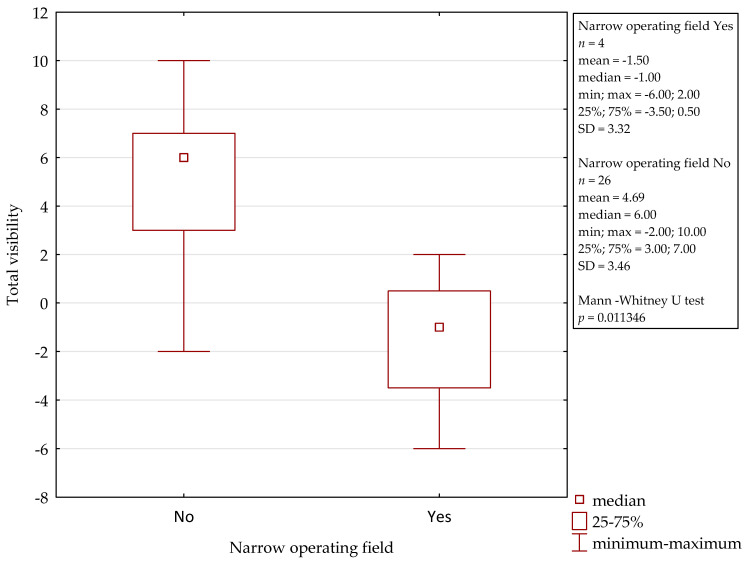
Influence of the narrow operative field on the visibility with the 3D exoscope during tympanoplasty.

**Figure 7:**
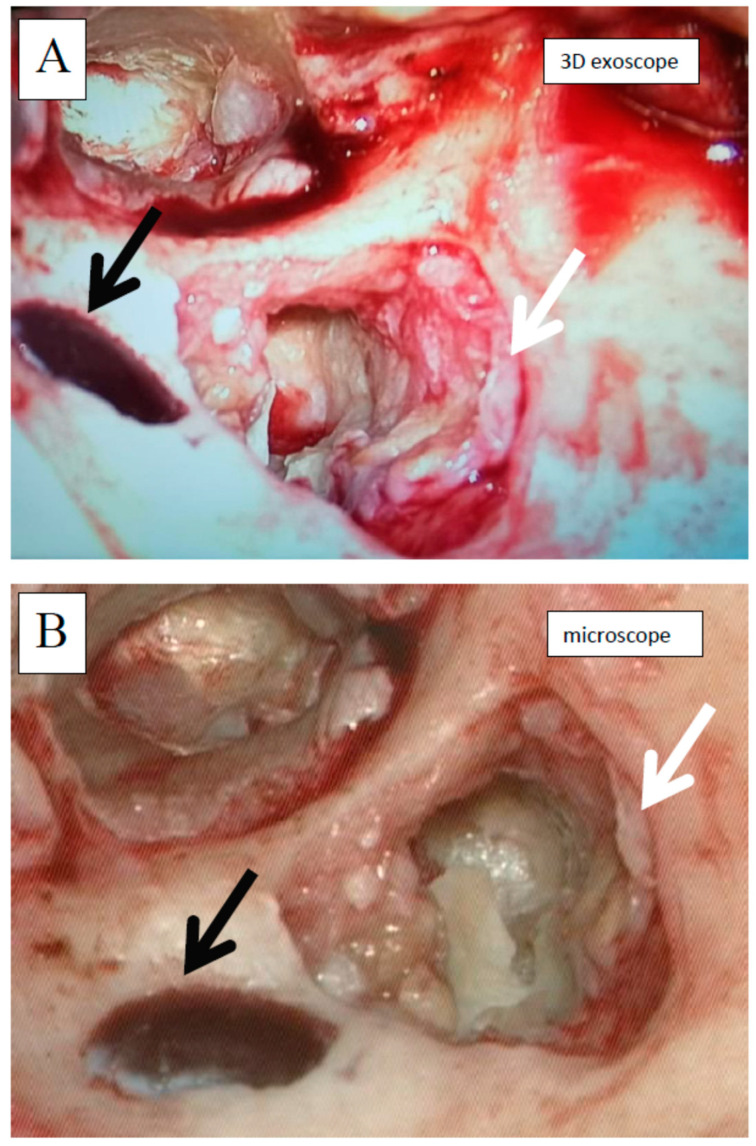
Intraoperative view during cholesteatoma surgery (left ear, retroauricular approach); antromastoidectomy performed, cholesteatoma partially removed, cholesteatoma matrix visible (white arrow), sigmoid sinus partially exposed (black arrow); **A**—with the exoscope, **B**—with the microscope. Images shown are 2D representations.

In comparison to the microscope, the 3D exoscope was evaluated as better in 56.3%, as comparable in 26.0%, and as worse in 17.7% for the whole procedure. Differences were statistically significant (*p* = 0.0283). The sum of the obtained points for all the stages of all the surgeries was 116 with a mean of 3.87 and a median 5.00 for one complete procedure. There were no significant differences in the surgical field visibility for the first and second cholesteatoma surgeries. However, we found some evident differences concerning the influence of intraoperative conditions. Both intraoperative bleeding (observed in six cases) and the narrow surgical field (found in another four cases) significantly reduced the visibility with the 3D exoscope in comparison to the microscope. The details are presented in Figure 5 and Figure 6. An intraoperative view during cholesteatoma surgery, both with the exoscope and the microscope, is presented in Figure 7.

## 4. Discussion

The aim of the present study was to describe the first experience with the high-definition 3D exoscope for the surgical treatment of otosclerosis and chronic otitis media. Our findings show that nearly all the procedures were completed successfully (without any complications) using the 3D exoscope, which provided sufficient visualization of crucial anatomical structures. Conversion to the operating microscope was necessary in only two cases (3.3%), both during cholesteatoma or granulation removal from tympanic cavity and hidden areas of the middle ear (protympanum and/or sinus tympani). These findings support the use of the 3D exoscope for middle ear surgery. Based on our findings, the 3D exoscope appears to represent a valid 3D stereoscopic visualization tool for use in middle ear surgery. Our study showed that exoscope is an excellent tool for the more lateral part of the middle ear but has some disadvantages for surgery medial to the tympanic membrane.

The main advantages of the exoscope include high-resolution 3D images, a greater degree of freedom for the exoscope adjustment, and a comfortable surgical posture [12,13]. What is more, in our sample, the 3D exoscope did not require more surgical time than the operating microscope.

However, based on our subjective observations, the 3D exoscope has some important limitations, both during stapedotomy and tympanoplasty. In both procedures, the superiority of the exoscope over the microscope was only observed during more superficial stages. By contrast, in deeper areas of the middle ear, as the surgical field narrows and bigger magnification is required, the exoscope provided statistically significantly worse visibility. During stapedotomy, all the steps in the oval window region (S7–S10) were visible better with the microscope. Similarly, during tympanoplasty, we found that the microscope was more comfortable when removing the cholesteatoma or granulation tissue from the tympanic cavity and hidden areas of the middle ear (stages T6 and T7). Similar observations in microvascular bypass procedures have been reported [14]. As those studies have shown, microvascular anastomosis is feasible under 3D exoscope visualization; however, at the highest magnification, the depth perception is inferior to that provided by a standard operating microscope, which impedes the procedure [14]. In our opinion, the visualization of deeper structures with the exoscope was not ideal as the system relies solely on digital zoom. Thus, the lack of optical zoom was a cause of the deterioration of the surgical image when using higher magnification. This limitation, in our opinion, is especially important for less experienced otosurgeons, who would not be able to complete the procedure under worse quality of visibility.

Another limitation of visualizing the surgical field with the 3D exoscope is the narrow operating field and bleeding during the surgery, cases in which a microscope is preferable. The presence of these unfavorable factors in the operative field had a significant and negative impact on the quality of the visibility provided by the 3D exoscope in comparison to the microscope. Thus, in such cases, we strongly advise that a microscope be chosen rather than the 3D exoscope.

### Study Limitations and Strengths

This study has several limitations. Firstly, the operating surgeons compared the quality of visibility with the exoscope to their prior recollection of visibility from other surgeries with the microscope. Study design relies heavily on a subjective assessment of a surgeon in real time. Undoubtedly, achieving an unbiased fashion is very difficult in such a comparison. However, from a technical point of view, conducting surgery on the same patient by means of two instruments simultaneously (an exoscope and a microscope) was not possible. That is why a surgeon’s recollection, knowledge, and experience seemed to be an invaluable reference point. Secondly, we were unable to unify all steps of tympanoplasty. For this reason, to facilitate the comparison, we selected main surgical milestones for tympanoplasty. A third limitation is that we did not include data concerning body habitus (i.e., weight or difficulty of positioning), even though such data are important for the 3D exoscope application. One of the challenges of using a microscope is that visualization may be limited, and a surgeon’s operative comfort decreases when the patient’s body habitus or anatomy is unfavorable. The next limitation of our research is the lack of postoperative hearing test results. This is due to the initial boundary assumption that the results must be as good as in cases of the classical method. Therefore, in cases of difficulties in visualization with the exoscope and suboptimal insight, we were immediately ready to convert the operation to the microscope to avoid complications or a worse audiological result. Finally, the last limitation is our inability to include 3D photographs in this manuscript, as one of the major aspects of the comparison between the use of the 3D exoscope and a microscope involved the comparison of the quality of 3D images. By contrast, the main strength of this study is the fact that, to our knowledge, it was the first study to assess the value of the 3D exoscope in stapedotomy and tympanoplasty. In addition, the analysis was carried out by a reference center with a 40-year tradition in otosurgery that performs approximately 900 procedures per year.

## 5. Conclusions

Overall, our study shows that the 3D exoscope offers excellent, highly magnified, and well-illuminated high-definition images of the surgical field. However, our experience revealed several important limitations of this system, including decreased depth perception in deep areas of the tympanic cavity and reduced visibility in a difficult surgical field, with the subsequent need to switch to an operating microscope in select cases.

## Figures and Tables

**Table 1:** The visibility of the surgical field in stapedotomy by stage: the 3D exoscope versus the microscope.

		Visualization Grade: 3D Exoscope Relative to Microscope				
Stapedotomy—Stages of the Procedure		WORSE (−1)	COMPARABLE (0)	SUPERIOR (1)	SCORE (Sum of Points)	*p*-Value (Test of Proportion)
S1	Initial view of the operating field	0 (0.0%)	4 (13.3%)	26 (86.7%)	26	0.0001
S2	Tympanomeatal flap formation	4 (13.3%)	0 (0.0%)	26 (86.7%)	22	0.0001
S3	Tympanic cavity opening	4 (13.3%)	4 (13.3%)	22 (73.4%)	18	0.0104
S4	External auditory canal widening	4 (13.3%)	3 (10.0%)	23 (76.7%)	19	0.0034
S5	Incudostapedial joint visualization	7 (23.3%)	4 (13.3%)	19 (63.4%)	12	0.1421
S6	Oval window area assessment	7 (23.3%)	6 (20.0%)	17 (56.7%)	10	0.4630
S7	Stapes superstructure removal	18 (60.0%)	8 (26.7%)	4 (13.3%)	−14	0.0001
S8	Facial nerve assessment	16 (53.3%)	11 (36.7%)	3 (10.0%)	−13	0.0001
S9	Footplate assessment and perforation	20 (66.7%)	8 (26.7%)	2 (6.6%)	−18	0.0001
S10	Prosthesis placement maneuver	17 (56.7%)	11 (36.7%)	2 (6.6%)	−15	0.0001
Stapedotomy—the whole procedure (sum of S1–S10)		97 (32.3%)	59 (19.7%)	144 (48.0%)	47	0.4884

**Table 2:** The visibility of the surgical field in tympanoplasty by stage: the 3D exoscope versus the microscope.

		Visualization Grade: 3D Exoscope Relative to Microscope				
Tympanoplasty—Stages of the Procedure		WORSE (−1)	COMPARABLE (0)	SUPERIOR (1)	SCORE (Sum of Points)	*p*-Value (Test of Proportion)
T1	Initial view of the operating field—planum mastoideum	0 (0.0%)	6 (20.0%)	24 (80.0%)	24	0.0010
T2	Posterior meatal wall skin incision + tympanic membrane assessment	3 (10.0%)	6 (20.0%)	21 (70.0%)	18	0.0285
T3	Antromastoidectomy	1 (3.3%)	7 (23.3%)	22 (73.4%)	21	0.0104
T4	Cholesteatoma or granulation removal from mastoid process	5 (16.7%)	5 (16.7%)	20 (66.7%)	15	0.0673
T5	Lateral semicircular canal visualization	2 (6.7%)	8 (26.7%)	20 (66.7%)	18	0.0673
T6	Cholesteatoma or granulation removal from tympanic cavity	18 (60.0%)	9 (30.0%)	3 (10.0%)	−15	0.0001
T7	Cholesteatoma or granulation removal from hidden areas (protympanum and/or sinus tympani)	19 (63.3%)	9 (30.0%)	2 (6.7%)	−17	0.0001
T8	Temporal fascia harvesting	0 (0.0%)	7 (23.3%)	23 (76.7%)	23	0.0034
T9	Ossiculoplasty	2 (6.7%)	12 (40.0%)	16 (53.3%)	14	0.7172
T10	Myringoplasty	3 (10.0%)	9 (30.0%)	18 (60.0%)	15	0.2733
Tympanoplasty—the whole procedure (sum of T1–T10)		53 (17.7%)	78 (26.0%)	169 (56.3%)	116	0.0283

## Data Availability

The data presented in this study are available on request from the corresponding author.
